# Reply to Amarin et al., “Selection bias may compromise our understanding of the clinical significance of the co-detection of respiratory viruses”

**DOI:** 10.1128/spectrum.01896-24

**Published:** 2024-09-19

**Authors:** Kim Stobbelaar, Benedicte Y. De Winter, Annemieke Smet, Stijn Verhulst, Peter L. Delputte

**Affiliations:** 1 Faculty of Medicine and Health Sciences, University of Antwerp, Antwerp, Belgium; 2 Laboratory of Microbiology, Parasitology and Hygiene, University of Antwerp, Antwerp, Belgium; 3 Laboratory of Experimental Medicine and Pediatrics, University of Antwerp, Antwerp, Belgium; 4 Department of Pediatrics, Antwerp University Hospital, Edegem, Belgium; 5 Infla-Med Centre of Excellence, University of Antwerp, Antwerp, Belgium; 6 Department of Gastroenterology and Hepatology, Antwerp University Hospital, Edegem, Belgium; Barnard College, Columbia University, New York, New York, USA

## REPLY

First, we would like to thank Amarin et al. for their interest in our paper. The concern was raised that our study population, being medically attended Respiratory Syncytial Virus (RSV) infections in children below the age of 2, does not accurately represent the whole population susceptible to RSV infection. Although we completely agree with this statement, we have never claimed such representativeness. In fact, we explicitly stated not to draw strong conclusions about the general population and identified the low number of non-hospitalized patients as a limitation in our discussion.

To allow for a valid inference about the whole population, we acknowledge that, ideally, community data are gathered simultaneously to serve as a reference point for studies performed in a hospital setting. The complexity of obtaining such data in young and vulnerable populations must, however, be recognized. Furthermore, it is important to note that “medically attended” does not necessarily correlate with increased disease severity. This is exemplified by the median ReSViNET score of 8 (IQR: 5–11.25) in our study population, which corresponds to moderate disease severity.

We agree with the statement that RSV is generally more virulent than other respiratory viruses. However, this seemingly contradicts the possibility of RSV being a secondary driver of infection in most patients within our co-infection group. With a few exceptions (*n* = 8/47), patients in this co-infection group exhibited lower ct values for RSV (mean = 22.3, SD = 5.7) compared to the co-detected viruses (mean = 29.0, SD = 5.7). This suggests that RSV is the main driver of symptomology at the moment of sampling for the majority of patients with co-infections, although some regression to the mean can, indeed, not be ruled out.

Taken together, while we acknowledge the potential for selection bias in studies of this nature, we wish to reiterate that our aim was to underscore the complexity and multifactorial cause of the differential disease severity observed in RSV-infected infants and the possible role co-infections might play in this. In our study of medically attended RSV infections, the conclusion seems to be that more severe disease is observed in patients in which only RSV was detected.

